# How the Ecdysozoan Changed Its Coat

**DOI:** 10.1371/journal.pbio.0030349

**Published:** 2005-10-11

**Authors:** John Ewer

## Abstract

External skeletons are found in a variety of animals, including arthropods and nematodes. Much remains to be learned about the process of replacing the exoskeleton (molting) during growth.

Most people are aware that insects are crunchy on the outside. What they don't always realize is that the external crunchy parts form the animal's skeleton, and that this body design makes the apparently simple process of growing extremely complex.

In addition to providing a barrier against desiccation and protection from mechanical injury, an insect's external skeleton (exoskeleton) does what skeletons do for all animals: it gives the insect its shape and also provides the frame to which muscles are attached, and, therefore, is critical for its behaviors. External skeletons are found in a variety of animals, including arthropods (“jointed-foot invertebrates,” including insects, crustaceans, spiders, millipedes, and centipedes) and nematodes. Arthropods include some of the most successful organisms on earth. Ants alone are believed to represent up to one-third of the terrestrial animal biomass [1], and as many as half of earth's species may be insects.

The undeniable success of insects is most certainly due, in part, to the group's rugged exoskeleton. Nevertheless, living within an external skeleton immediately raises a logistical problem: how to grow. Although many insects, especially at immature stages, have elastic skeletons, continuous growth eventually requires that the skeleton be replaced with a larger one. The process that effects this replacement is called molting, and although the same term is used to describe the replacement of the outer skin layer of some vertebrates such as snakes, the process of replacing an exoskeleton is infinitely more difficult. The ability to replace an exoskeleton is currently believed to have evolved only once during animal evolution, giving rise to a clade of animals called Ecdysozoa, which includes arthropods and nematodes [2].

## The Mechanics of Molting

The exoskeleton, or cuticle, is a well-defined inert structure that is secreted by, and strongly attached to, the underlying epidermal cells. Although its composition varies significantly among ecdysozoans (e.g., consider the skeleton of a beetle versus that of a crab), the process of molting itself is similar within the clade: the epidermis may undergo a round of cell division (thereby producing a larger surface) and separates from the exoskeleton. A new exoskeleton is then secreted by the epidermis, but is soft until the remains of the overlying old cuticle are shed at ecdysis. The new cuticle then expands and hardens.

In nature, this process appears simple only because ecdysozoans are experts at molting. But underlying this seamless process is a complex developmental feat, as the animal must replace its skeleton and survive the process in an unsheltered environment. For instance, despite the erosion of the cuticle's integrity that takes place during an insect molt, muscle attachments on the old cuticle remain functional until that cuticle is shed, at which time the corresponding attachments on the new cuticle are fully differentiated. Since the new cuticle is soft until it is sclerotized, rigidity is initially aided by an increase in internal pressure caused by swallowing of water or air at the time of ecdysis (shedding of the old cuticle). Like insects, nematodes are able to move during the lethargus that accompanies the molt, even though the old cuticle separates from the epidermis and muscle attachments are likely remodeled during this time. In insects, the sensory bristles attached to the cuticle remain functional until shortly before they are shed along with the rest of the exoskeleton. Then, over the course of just a few minutes, innervation by the same sensory neuron is transferred to the new bristle located on the underlying new cuticle. Finally, the lining of the main air ducts (trachea), which form a complex web of tubes that extends throughout the body, also molts. During this process, air exchange is likely compromised because of the presence of a new layer of developing cuticle and the molting fluid that is present between the old and new cuticles, which aids in the digestion of the old exoskeleton. While this complex process is taking place, the insect remains a potential prey, and is also prone to dehydration, since the old cuticle is weakened and the new cuticle has not yet been rendered impermeable by tanning. These considerations (and others) place tremendous constraints on the timing and choreography of the molting process.

Despite the complex internal upheaval, molting does not typically affect the insect's performance until the very end of the molt, when the remains of the old cuticle are shed at ecdysis and the new cuticle rapidly expands and hardens. It is mostly during this brief period that the animal is vulnerable: once the behavior of ecdysis starts, it cannot be interrupted, and even minor hitches can be fatal. For instance, failures to fully extricate a wing may cause a malformed wing to be hardened, forever compromising the adult's ability to fly.

This short list highlights some of the processes that must occur perfectly at every molt (some insects molt hundreds of times during their lifetime), and does not even include those processes for the much more complex process of insect metamorphosis. During a metamorphic molt the cuticle is also replaced; however, the whole animal is essentially redesigned at this time as it transforms from a larva to an adult. The different shape, appendages, sensory and motor capabilities, and diet of a caterpillar versus a butterfly give some idea of the magnitude of the changes that take place during metamorphosis. Nevertheless, a “simple” molt is complex enough, as the exoskeleton must be replaced as quickly as possible, and produce a functional animal when completed, with a cuticle that permits feeding and fleeing, and that provides a barrier to desiccation.

## Molecular Control of Molting in Arthropods

Our understanding of the control of molting in insects stems from simple, clever experiments carried out in the early part of the 20th century by the founders of insect endocrinology, including Kopéc, Wigglesworth, and others [3]. Using parabiosis (the experimental joining of two organisms for the purpose of studying the effects of one on the other), ligatures, and transplants, they showed that molting and metamorphosis are controlled and coordinated by systemic hormonal signals. Thus, for example, Wigglesworth showed that molting could be induced in a nonmolting insect by joining it to an insect that was in the process of molting. Since the two insects only shared a circulatory system, this result indicated that molting was induced by a circulating hormone, now known to be the steroid 20-hydroxyecdysone (20E).

We also know that insect molting is initiated by the secretion of a brain neuropeptide called prothoracicotropic hormone in response to poorly understood signals that integrate the animal's size, weight, nutritional status, as well as photoperiodic information. This peptide acts on the peripheral prothoracic gland, causing it to synthesize and secrete the steroid hormone ecdysone (E). E is then converted by peripheral tissues to the active molting hormone, 20E.

20E is the key hormone that regulates the molt. The increase in 20E titers initiates the molt. For instance, it causes the epidermis to divide, separate from the old cuticle, and synthesize and secrete new cuticle components. The decrease in 20E titers at the end of the molt allows the molt to be completed. In particular, this fall is required to set in motion a complex hierarchy of interacting neuropeptides, which act on the nervous system to initiate ecdysis behaviors and on the peripheral tissues to the hardening of the new cuticle, among other peripheral actions [4].

20E acts mostly on members of the conserved nuclear receptor superfamily, which act as ligand-dependent transcription factors. Thus, 20E activates the complex transcriptional processes that underlie the many cellular and morphogenetic events that occur during molting. Being a circulating lipid-soluble (steroid) hormone gives 20E a built-in ability to affect all cells in the organism and cause widespread changes. Such coordination is critical for a successful molt, and is mostly effected by each tissue responding autonomously to the changes in 20E titers, with little tissue-to-tissue coordination. Although much more complex than “simple” molts, metamorphic molts are also directed by 20E. This global endocrine signal is then transduced into a very complex, tissue-specific pattern of gene expression by turning on (and off) 20E-regulated genes, which in turn activate or repress other genes in the pathway that eventually turns a caterpillar into a butterfly [5].

Comparatively less is known about how other ecdysozoans change their coat. Work in crustacea has established that a steroid hormone also controls molting within this class [6]. In fact, many crustacea produce E, which is then converted to the active hormone 20E, the exact same hormone that controls insect molting. Little is know about the mechanism that causes the onset of the molt and the downstream actions of 20E in crustacea, but there are bound to be interesting similarities as well as differences compared to the process in insects. For instance, we already know that 20E production in crustacea is regulated by a neuropeptide (molt-inhibiting peptide) that inhibits E production, rather than by a neuropeptide that stimulates E synthesis (as occurs in insects).

## Molting in Worms

Relatively little is known about the process of molting in nematodes. However, this group of organisms is significant for several reasons. First, nematodes are quite different from arthropods; yet their membership in the ecdysozoan clade reflects a deep biological relatedness. Comparisons between molting in nematodes and molting in arthropods would reveal, for instance, which features are invariant (and presumably very constrained) aspects of molting, and which are different solutions to the same problem. Second, a number of serious human diseases are caused by parasitic nematodes. Agents that interfered with molting would be fatal to the parasite, but may not affect its host, to which the parasite is only very distantly related (“A worm, with very few exceptions, is not a human being” [7]). Finally, the nematode Caenorhabditis elegans is a model organism, with a sequenced genome and well-developed molecular genetic tools. These tools make it possible to carry out forward and reverse genetic screens, determine the time and place that candidate genes are expressed, investigate order of gene action, etc., and have been used to systematically analyze many aspects of the biology of this little worm.

Paradoxically, until now, molting has not been subjected to such systematic scrutiny. This may be because recognition of nematodes' membership in the ecdysozoan club is recent, so molting has not been viewed as a prominent feature of their biology. Nevertheless, a number of known features of molting in C. elegans make it especially intriguing. For instance, it has been difficult to demonstrate that molting in nematodes requires cholesterol, the precursor of steroid hormones, and C. elegans lacks key enzymes required for the synthesis of sterols [8]. In addition, despite encoding a bounty of 260 nuclear receptors, the C. elegans genome does not apparently encode a homolog of the 20E receptor or of *ultraspiracle*, its obligate partner.

**Figure 1:**
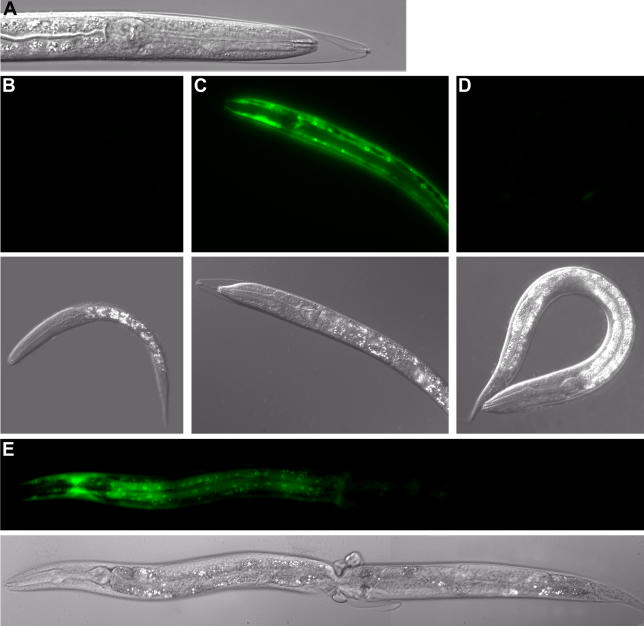
A C. elegans Molting Sampler (A) A C. elegans larva unable to shed its old cuticle after RNA interference of the gene *acn-1*. (B–D) Pairs of pictures show larvae expressing the GFP-tagged *mlt-11* gene before (B), during (C), and after (D) the first molt. (E) A collar of old cuticle found midway along the length of the animal mostly restricts expression of the GFP-tagged *mlt-10* gene to the front half of the worm.

The research article by Frand et al. [9] in this issue of *PLoS Biology* reports the results of the first systematic screen for genes involved in molting in nematodes. The authors used RNA interference to silence each of the 19,427 predicted C. elegans genes, and screened for the occurrence of worms that were unable to shed their old cuticle (Figure 1A). This screen produced a list of 159 genes whose interference produced molting defects in larvae. Significantly, interference with gene function could cause arrest at different larval molts, suggesting that many of these genes are required for molting, per se, not simply for the transition between two specific stages. Importantly, the 159 genes included nine genes that had previously been shown to be involved in C. elegans molting, as well as 28 that had previously been noted as causing an arrest during a molt when inactivated by RNA interference. The nine genes include *nhr-25*, the homolog of the orphan nuclear receptor *ftz-f1* [10], which plays a key role in insect metamorphosis, as well as *lpr-1* [11], a homolog of *gp330-megalin*, which is involved in vitamin D (a cholesterol derivative) uptake in mammals. Re-isolating these genes in a forward screen for molting defects lends further support to the (wishful) hypothesis that steroid hormones are involved in nematode molting, just as they are in molting in the arthropod members of this clade. Although the screen was based on a failure in the last step of the molt (shedding of the old cuticle), many of the candidates are likely to play a role in the earlier stages of the molting process. Indeed, the list of genes includes transcription factors, signaling proteins, and molecules involved in cuticle secretion and remodeling of basement membranes, as well as the expected list of proteins involved in the eventual release of the collagenous cuticle.

As expected for a first description of the results of a genome-wide screen, it is not yet possible to weave all the candidate genes into a solid story. Nevertheless, the authors do make a significant contribution to our understanding of how these genes might make the nematode change its coat by investigating in more detail eight genes representing the major functional categories. First, fusions to green fluorescent protein (GFP) revealed that all eight genes were expressed in epithelial cells, with a pulse of fluorescence prior to each molt (Figure 1B–1D). Thus, these genes are expressed in the right place at the right time. Second, the ability of RNA interference of molting genes to block GFP expression of the select tagged molting genes was used to order gene expression cascades. This analysis revealed, for instance, that interference of *nhr-23* (encoding another orphan nuclear receptor previously implicated in molting [12]) affected the expression of the eight GFP-tagged molting genes, suggesting that *nhr-23* may be generally required for the expression of molting genes. By contrast, blocking the molting gene *mlt-9* caused the expected molting defect without affecting the expression of *mlt-10* and *mlt-8*. As an additional bonus from this study, the authors noted that some genotypes tended to develop constrictions in their cuticle, and that in these animals, both GFP expression and molting were confined to the anterior part of the animal (Figure 1E). This result is reminiscent of the now classic experiments in insect endocrinology that demonstrated the role of circulating hormones in the control of molting [3]. Thus, Frand et al., in a single study, have carried out classic and molecular genetic experiments to provide a qualitative advance in our understanding of how nematodes molt.

The information gained from this screen is important in itself, since few genes involved in nematode molting had previously been identified. These genes can now be compared to the homologous genes in other ecdysozoans, and may also be useful for developing effective agents for controlling parasitic nematodes. Furthermore, modifications of the screen that was used (e.g., sensitized screens) may allow the nematode to answer some of the more intractable questions regarding the control of molting. For example, little is known about the mechanism that monitors the organism's size and signals that a molt must start. In insects, we know that it is dependent on size (weight), nutritional status, and time of day, but how this information is integrated and transduced is unknown for most species. Likewise, little is known about how ecdysozoans know how many immature stages they must complete before becoming an adult. For example, we do not know how a third-instar caterpillar determines that it still has two more caterpillar stages to go through before becoming a pupa (chrysalis), how a third-instar fruit fly larva knows that it has completed its larval life, and how a third-instar C. elegans knows that it still has a fourth instar to go through before becoming an adult. An interesting possibility is that the process of “instar counting” is regulated by so-called heterochronic genes [13]. Thus, is a failure to enter metamorphosis (for an insect) mechanistically related to adding an extra larval instar (for a worm)? The screen conducted by Frand et al. did not identify any known heterochronic genes, but this could simply reflect the criteria used for identifying candidate genes. Finally, we know little of both global checkpoints that must be cleared in order for molting to progress and any signals between molting tissues. Frand et al.'s research article paves the way for a new understanding of molting in Ecdysozoa.

## Abbreviations

20E: 20-hydroxyecdysone
E: ecdysone
GFP: green fluorescent protein
